# Serum 25-hydroxyvitamin D and parathyroid hormone in relation to plasma B-type natriuretic peptide: the Hoorn Study

**DOI:** 10.1530/EC-12-0033

**Published:** 2012-07-21

**Authors:** Adriana J van Ballegooijen, Marjolein Visser, Marieke B Snijder, Jacqueline M Dekker, Giel Nijpels, Coen D A Stehouwer, Michaela Diamant, Ingeborg A Brouwer

**Affiliations:** 1 Department of Health Sciences, Faculty of Earth and Life Sciences EMGO^+^, Institute for Health and Care Research, VU University Amsterdam De Boelelaan 10851081 HV, Amsterdam The Netherlands; 2 Department of Epidemiology and Biostatistics EMGO^+^, Institute for Health and Care Research, VU Medical Center Amsterdam The Netherlands; 3 Department of Public Health Academic Medical Center, University of Amsterdam Amsterdam The Netherlands; 4 Department of General Practice VU Medical Center Amsterdam The Netherlands; 5 Department of Internal Medicine and Cardiovascular Research Institute Maastricht Maastricht University Medical Centre Maastricht The Netherlands; 6 Department of Internal Medicine Diabetes Cente, VU Medical Center Amsterdam The Netherlands

**Keywords:** vitamin D, parathyroid hormone, B-type natriuretic peptide, epidemiology

## Abstract

**Objective:**

A disturbed vitamin D–parathyroid hormone (PTH)–calcium axis may play a role in the pathogenesis of heart failure. Therefore, we investigated whether lower 25-hydroxyvitamin D (25(OH)D) and higher PTH are cross sectionally and after 8 years of follow-up associated with higher B-type natriuretic peptide (BNP) levels in older men and women.

**Design and methods:**

We measured baseline 25(OH)D, PTH, and BNP in 502 subjects in 2000–2001 in the Hoorn Study, a population-based cohort. Follow-up BNP was available in 2007–2009 in 278 subjects. Subjects were categorized according to season- and sex-specific quartiles of 25(OH)D and PTH at baseline. We studied the association of 25(OH)D and PTH quartiles with BNP using linear regression analyses adjusting for confounders. Analyses were stratified by kidney function estimated glomerular filtration rate (eGFR; ≤60 ml/min per 1.73 m^2^) because of significant interaction.

**Results:**

At baseline, subjects had a mean age of 69.9±6.6 years, mean 25(OH)D level was 52.2±19.5 nmol/l and mean PTH 6.1±2.4 pmol/l. Cross sectionally, 25(OH)D was associated with BNP in subjects with impaired kidney function (eGFR ≤60 ml/min) only. The association attenuated after adjustment for PTH. PTH was cross sectionally associated with BNP, also in subjects with impaired kidney function only: regression coefficient of highest quartile 9.9 pmol/l (95% confidence interval 2.5, 17.4) with a significant trend across quartiles. Neither 25(OH)D nor PTH was associated with BNP in longitudinal analyses.

**Conclusion:**

This study showed overall no strong association between 25(OH)D and BNP. However, PTH was associated with BNP in subjects with impaired kidney function and may point to a potential role in myocardial function.

## Introduction

Worldwide, vitamin D deficiency is highly prevalent among older people (1). Vitamin D can be either ingested orally or can be synthesized by the human skin by exposure to the solar ultraviolet B spectrum (1). Substantial decrease in serum 25-hydroxyvitamin D (25(OH)D) levels, the principal circulating storage form, will result in proportionally higher parathyroid hormone (PTH) levels to maintain serum and total body calcium. Vitamin D and PTH receptors have been detected in (skeletal and in) cardiac muscle (2, 3), indicating a potential functional role in myocardial function. Thereby, a disturbed vitamin D–PTH–calcium axis – which includes calcitriol the active form of vitamin D that is produced in the kidney – may play a role in the pathogenesis of heart failure (HF) (4).

Loss of heart function or HF is a growing problem in our aging society, therefore determination of factors that can influence loss of heart function is of paramount importance. Recent evidence suggests a number of mechanisms where 25(OH)D and PTH may influence the pathophysiology of HF. These include actions on blood pressure via the renin–angiotensin system, calcium handling, reduction of proinflammatory cytokines, left ventricular mass, and improvements in endothelial function (4, 5, 6).

Plasma B-type natriuretic peptide (BNP) is a neurohormone that is primarily secreted in the cardiomyocytes in response to ventricular stretch and pressure overload, so the BNP level is determined by many aspects of cardiac function (7). Subjects with BNP levels below 100 pg/ml (equivalent to 28 pmol/l) have no symptomatic HF. However, in asymptomatic subjects, higher BNP levels – but still below the threshold of 100 pg/ml – indicate a higher future risk of developing HF, atrial fibrillation, stroke, transient ischaemic attack, and death (8). This makes BNP a global marker for myocardial deterioration.

Patients with elevated BNP levels have often normal cardiac function when measured by conventional echocardiography but suffer from preclinical systolic and diastolic dysfunction as assessed by tissue Doppler imaging (9). Circulating levels of NT-proANP – released by cardiomyocytes in the atria – are largely increased in patients with HF and are a predictor of disease severity (10). This suggests that a moderate increase in plasma BNP level may serve as a sensitive marker for early myocardial deterioration (8, 9), which cannot be detected with conventional echocardiography. The relationship between 25(OH)D and PTH in relation to kidney function is well-known, whereas the relationship with BNP is novel (11).

Observational data in patients with HF suggest that low 25(OH)D levels and high PTH levels are common and are both associated with higher BNP levels (12, 13). This indicates that ventricular stretch due to pressure overload is the primary driver of the secretion of BNP in the cardiomyocytes and represents a long-term indirect measure of cardiac function. So far, there are no observational data available for 25(OH)D and PTH in relation to BNP levels in a general population. We therefore tested the hypothesis whether lower 25(OH)D levels and higher PTH levels are associated with higher BNP levels in older men and women, both cross sectionally and after 8 years of follow-up.

## Materials and methods

### Study sample

The Hoorn Study started in 1989 and included 2483 men and women aged 50–75 years, from the Netherlands. Detailed descriptions of the study concept and baseline measurements have been published previously (14). The current study was done in 648 subjects who had examinations in 2000–2001 (considered as baseline) (15). Subjects with no assessment of PTH (*n*=15) or 25(OH)D (*n*=26) were excluded. Subjects with no baseline BNP measurement were also excluded (*n*=105). This resulted in 502 subjects with vitamin D, PTH, and BNP measurements for cross-sectional analyses.

In 2007–2009, ∼8 years after baseline, 169 subjects were not willing to participate for the follow-up examinations. We excluded subjects with no BNP measurement at follow-up (*n*=55). Our study sample consisted of 278 subjects for longitudinal analyses. The study complied with the Declaration of Helsinki, the local Ethics Committee approved the study, and all subjects provided written informed consent.

### Blood measurements

Vitamin D status was assessed by measuring serum 25(OH)D levels by a competitive binding protein assay (DiaSorin, Stillwater, MN, USA) (16), a valid marker to measure vitamin D status (17). The inter-assay coefficient of variation (CV) was 10–15% (16). Serum intact PTH was determined by an immunoradiometric assay (DiaSorin, Stillwater, MN, USA). Laboratory measurements were conducted at the VU University Medical Center, Amsterdam.

Plasma BNP was determined in spare frozen EDTA samples that had been stored at −80 °C for 4 years. At baseline, BNP was determined using an immunoradiometric assay (Shionoria, Osaka, Japan). Inter- and intra-assay variability coefficients were analyzed within the normal range <10%. At follow-up, BNP was determined in spare frozen EDTA samples that had been stored at −80 °C for 2 years using a noncompetitive immunofluorometric assay (TRIAGE, Biosite, San Diego, CA, USA). Intra-assay CV was 3.5% and the total CV was 12.3%.

### Additional measurements at baseline

Fasting glucose, post-load glucose after an oral glucose tolerance test, high-density lipoprotein (HDL) and low-density lipoprotein cholesterol, and triglycerides were measured as described elsewhere (15). Weight (kg) and height (m) were measured while subjects were wearing light clothing and no shoes. Body mass index (BMI) was calculated as weight (kg) divided by height squared (m^2^). Waist circumference (cm) was measured at the level midway between the lowest rib and the iliac crest. Blood pressure (mmHg) was measured at the right-upper arm after 5 min at rest in a sitting position, using a random-zero sphygmomanometer (Hawksley–Gelma Ltd., Lancing, UK), and the average of two consecutive measurements was used. Arterial hypertension was defined as systolic blood pressure (SBP) ≥140 mmHg, diastolic blood pressure (DBP) ≥90 mmHg and/or use of antihypertensive medication. As an estimate of kidney function, glomerular filtration rate (eGFR) was calculated using serum creatinine values – determined using the Jaffé method – according to the Chronic Kidney Disease Epidemiology Collaboration (CKD-EPI) equation (18).

Subjects filled out questionnaires to obtain self-reported information on medication use, smoking status, education level, and physical activity. Current smoking was classified as yes/no. High education level was defined as higher vocational education or university, medium level as secondary education, and low level as elementary school, lower vocational training, or less. Physical activity (h/day), including commuting activities, leisure time activities, and occupational activities, was assessed by a validated questionnaire (16). Time spent on outdoor physical activity was estimated by adding the time spent on walking, cycling, and gardening.

### Statistical analysis

Baseline characteristics and BNP levels are presented according to sex- and season-specific 25(OH)D quartiles and PTH quartiles. Sex- and season-specific quartiles were used because of the described sex difference in 25(OH)D levels in our study (16) and absolute changes in 25(OH)D levels (19). We assume that serum 25(OH)D levels are relatively stable over a long time. A single 25(OH)D measurement can be used in epidemiological studies in which low 25(OH)D levels are linked to future diseases (20).

Season classification was based on the date of blood sampling: winter (December–February), spring (March–May), summer (June–August), and autumn (September–November). We used linear regression analyses to examine linear trends in the continuous variables across the quartiles of 25(OH)D and PTH using the categorical quartile variable as a continuous variable. A *χ* ^2^-test was performed to test for trend for categorical variables across the 25(OH)D and PTH quartiles.

We used multiple linear regression analyses to study the cross-sectional associations between baseline 25(OH)D quartiles and PTH quartiles in relation to baseline BNP levels. In the case of 25(OH)D, we considered the highest 25(OH)D quartile as the reference group, in the case of PTH we considered the lowest quartile as the reference group. We calculated a *P* value for trend by adding the 25(OH)D quartiles and PTH quartiles as a continuous variable to the regression models.

BNP values at baseline and at follow-up were determined by different methodologies. We added 9.2 ng/ml (equivalent to 2.66 pmol/l) to the baseline values of BNP as suggested by Hammerer–Lercher *et al*. (21). Differences between follow-up and baseline BNP were calculated to investigate changes within 25(OH)D and PTH quartiles.

Multiple linear regression analyses were used to study the association between baseline 25(OH)D quartiles and PTH quartiles and BNP levels after 8 years of follow-up. We adjusted for the baseline value by adding the baseline BNP as an independent variable to the regression models.

We adjusted for potential confounding in the regression models by adding potential confounders one by one to the regression models. Potential confounders considered were: waist circumference, BMI, outdoor activities, blood pressure, cholesterol, triglycerides, glucose levels, antihypertensive or lipid lowering medication use and eGFR. Variables that changed the regression coefficients of 25(OH)D or PTH quartiles more than 10% were included in the models. eGFR was added to describe separately the influence of adjustment for kidney function. In the case of PTH, we also adjusted for total 25(OH)D, because vitamin D deficiency is a known risk factor for hyperparathyroidism.

We tested for effect modification by presence of prior cardiovascular disease (CVD) because CVD treatment and self-care could change BNP. We also tested for effect modification by the presence of type 2 diabetes because subjects were invited according to their glucose tolerance status. Furthermore, we tested for interaction by presence of impaired kidney function (eGFR ≤60 ml/min per 1.73 m^2^) because lower kidney function may result in lower conversion of calcitriol (active form of vitamin D) and may increase PTH (22). When significant interaction terms were found (*P* value interaction term <0.10), analyses were stratified accordingly.

All reported *P* values were two-sided and values <0.05 were considered statistically significant. Statistical analyses were conducted using the statistical program PASW 17.0 (SPSS, Inc., Chicago, IL, USA).

## Results

Baseline serum 25(OH)D, PTH, and BNP were available for 502 subjects. Mean 25(OH)D level was 52.2±19.5 nmol/l and mean PTH was 6.1±2.4 pmol/l. Plasma BNP showed a distribution skewed to the right, median BNP was 7.7 pmol/l and interquartile range was 4.0, 11.3 pmol/l. The BNP residuals were normally distributed and therefore BNP is treated in its natural scale.

Baseline characteristics according to sex- and season-specific 25(OH)D quartiles showed that subjects with lower 25(OH)D levels were significantly older, were more often hypertensive, had less physical activities and outdoor activities, had higher BMI, larger waist circumference, higher blood pressure (SBP and DBP), higher post-load glucose levels, higher triglycerides, lower HDL cholesterol, higher PTH levels, and higher BNP levels with significant trends across the 25(OH)D quartiles (Table 1). Subjects in the third 25(OH)D quartile had on average the lowest BNP levels. Subjects with higher PTH levels were significantly older, had more often type 2 diabetes, were more often hypertensive, had higher BMI, larger waist circumference, higher blood pressure (SBP and DBP), higher fasting blood glucose, higher post-load glucose, lower HDL cholesterol, lower 25(OH)D levels, and higher BNP levels with significant trends across the PTH quartiles (Table 1).

### Cross-sectional analyses

**Figure 1:**
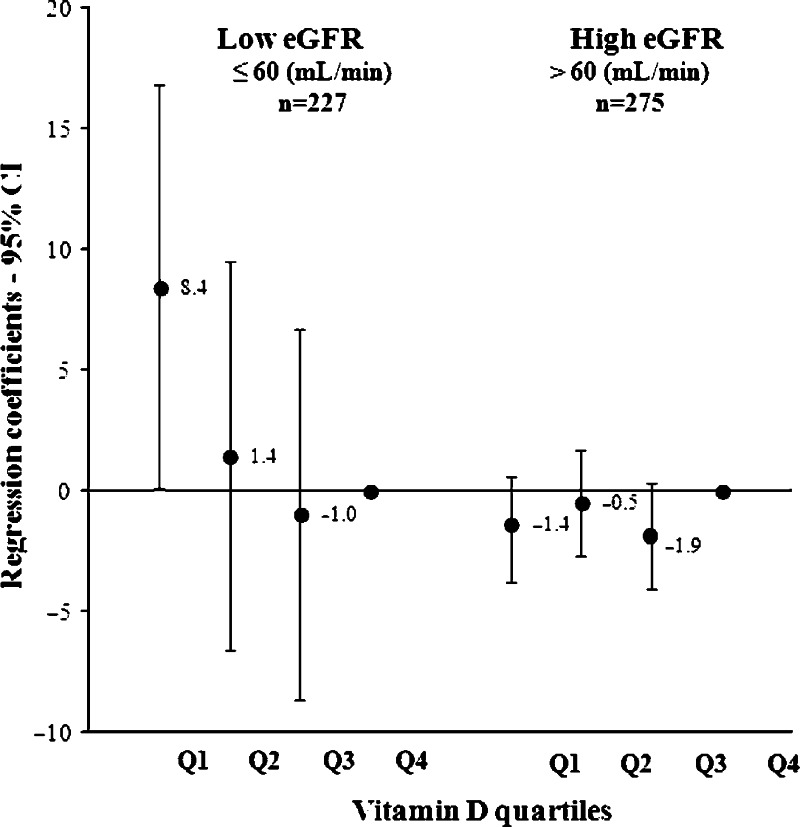
Regression coefficients of baseline 25(OH)D quartiles with B-type natriuretic peptide stratified by estimated glomerular filtration rate <60 ml/min in 502 older subjects. eGFR, estimated glomerular filtration rate; Q, quartile; Regression model: adjusted for age, smoking, outdoor activities, body mass index, triglycerides, HDL cholesterol. *P* for trend: impaired eGFR, 0.031; normal eGFR, 0.469.

We found effect modification by eGFR for the cross-sectional association of 25(OH)D with BNP (*P*<0.001) and not by prior CVD (*P*=0.162) or by the presence of type 2 diabetes (*P*=0.100). Therefore, we stratified the analyses based on kidney function: impaired kidney function (eGFR ≤60 ml/min per 1.73 m^2^) and normal kidney function (eGFR >60 ml/min per 1.73 m^2^). Stratification based on CKD stage is not possible because of too few subjects in the more severe stages of kidney function. Results of the linear regression analyses showed that in subjects with impaired kidney function the lowest 25(OH)D quartile was associated with higher BNP with a significant trend (*P*=0.031) across the 25(OH)D quartiles (Fig. 1). In other words, (on average) a 62% lower level of 25(OH)D resulted in a 82% higher BNP. Further adjustment for the residual kidney function – included as eGFR continuously – resulted in more pronounced results (*P* trend 0.013). In subjects with normal kidney function (eGFR >60 ml/min), no association was observed between 25(OH)D and BNP (Fig. 1) and also no association was found when 25(OH)D was treated as a continuous variable regression coefficient: 0.1 (−0.1, 0.1).

**Figure 2:**
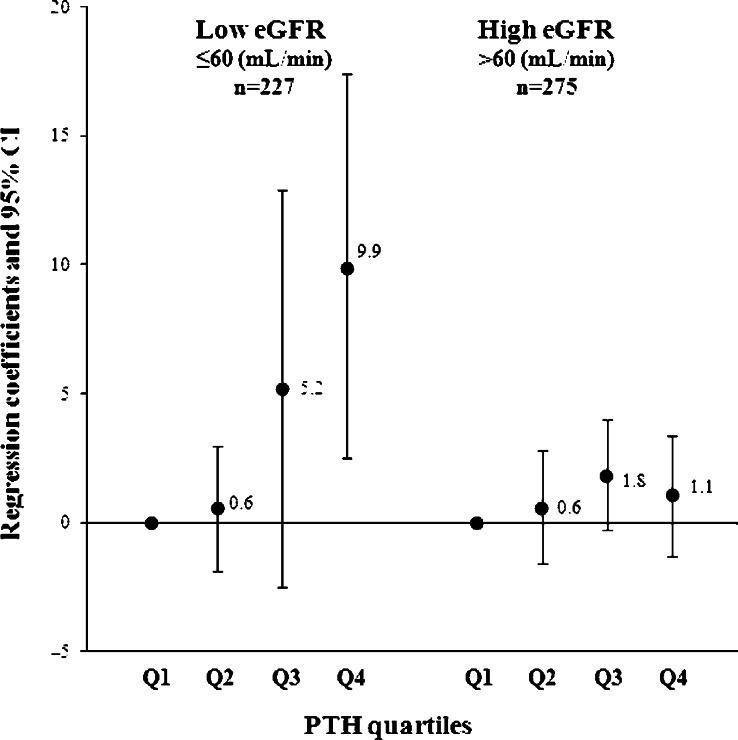
Regression coefficients of baseline parathyroid hormone quartiles with B-type natriuretic peptide stratified by estimated glomerular filtration rate <60 ml/min in 502 older subjects. eGFR, glomerular filtration rate; Q, quartile; Regression model: adjusted for age, smoking, outdoor activities, body mass index, triglycerides, HDL cholesterol; *P* for trend: impaired eGFR, 0.003; normal eGFR, 0.203.

We also found effect modification by eGFR for the cross-sectional association between PTH and BNP (*P*<0.001) but not by prior CVD (*P*=0.151) or by the presence of type 2 diabetes (*P*=0.171). In subjects with impaired kidney function (eGFR ≤60 ml/min), the highest PTH quartile was associated with higher BNP with a significant trend across the categories (*P*=0.003; Fig. 2). In other words, a 142% higher PTH level resulted in a 114% higher BNP. Further adjustment for kidney function attenuated the results slightly (*P* trend 0.007). The results attenuated only marginally and remained statistically significant when additionally adjusted for 25(OH)D with a significant trend across the PTH quartiles: regression coefficients were first quartile −1.5 (−9.2, 6.1), second quartile 4.0 (−3.7, 11.8), and third quartile 7.8 (0.0, 15.6; *P* trend 0.018). Similar results were seen when PTH was treated as a continuous variable: regression coefficient was 2.1 (1.1, 3.0) and changed slightly after adjusting for 25(OH)D, 1.9 (0.9, 2.9).

In subjects with normal kidney function, the association of PTH with BNP was in the same direction as for impaired kidney function although the association was less pronounced (Fig. 2). Regression coefficients were: first quartile 0.8 (−1.4, 3.0), second quartile 2.0 (−0.1, 4.2), and third quartile 1.3 (−1.1, 3.7; *P* trend 0.140).

### Longitudinal analyses

Baseline serum 25(OH)D, PTH, and baseline and follow-up BNP were available for 278 subjects. Mean 25(OH)D level was 54.7±18.4 nmol/l and mean PTH was 6.0±2.1 pmol/l. Median baseline BNP was 7.3 (interquartile range 4.1, 10.5) and median follow-up BNP was 14.5 (interquartile range 3.8, 25.2) pmol/l.

Also in this subsample, subjects in the lowest 25(OH)D quartile were significantly older, were more hypertensive, had less outdoor activities, larger waist circumference, higher blood pressure (SBP and DBP), and higher eGFR (data not shown). Subjects in the lowest 25(OH)D quartile had a significantly larger increase in BNP levels (Table 2). Subjects in the highest PTH quartile had a significantly higher BMI and larger waist circumference, higher DBP, and higher BNP. Changes in BNP did not differ significantly between PTH quartiles (Table 3).

Results of the longitudinal analyses in which follow-up BNP was adjusted for baseline BNP showed no association of 25(OH)D quartiles or PTH quartiles with BNP (Tables 2 and 3). We found no significant effect modification by prior CVD, type 2 diabetes, or eGFR for 25(OH)D and PTH in relation to 8 years changes in BNP.

## Discussion

In our study sample of older subjects, 25(OH)D was cross sectionally associated with higher BNP, only in subjects with impaired kidney function (eGFR ≤60 ml/min). The association was not independent of PTH, because adjusting for PTH attenuated the association. PTH was associated with higher BNP levels independent of 25(OH)D in subjects with impaired kidney function. In the longitudinal analyses, 25(OH)D and PTH were not associated with BNP levels after 8 years of follow-up.

The association between 25(OH)D and PTH in relation to cardiac markers such as BNP has not been studied extensively. In line with our study, cross-sectional studies showed that 25(OH)D was inversely associated with BNP levels in patients referred for coronary angiography (12, 23, 24). In randomized trials, a high dosage of vitamin D supplementation reduced BNP levels in type 2 diabetes patients (25) and HF patients (26). Cross sectionally, PTH was positively associated with BNP (13) in HF patients and not associated with BNP in patients on dialysis (24).

Our study is the first to investigate the association of 25(OH)D and PTH in relation to BNP in a general population. There were several reasons that could possibly explain why we were not able to show longitudinal associations. First, follow-up BNP measurements were determined using a different methodology than the baseline measurements. We applied a literature-based correction factor for baseline BNP, however, this may also introduce some bias. A second limitation is that missing data on follow-up BNP may not be missed at random but be related to conditions such as obesity or underlying cardiac abnormalities. Included subjects with a baseline and follow-up measurement were significantly younger (67.4 vs 69.7 years), had less hypertension (62 vs 67%), and lower waist circumference (93.1 vs 94.1 cm) than subjects who had no follow-up measurement. Therefore, the associations of 25(OH)D and PTH with BNP are possibly obscured because subjects with more cardiac abnormalities were lost to follow-up. Third, as an estimate of kidney function, eGFR was calculated with serum creatinine using the Jaffé method. Nowadays, the use of standardized creatinine measures is the standard for estimating GFR (18). This could have led to an overestimation of the amount of people with impaired kidney function (eGFR ≤60 ml/min). However, it did not influence the ranking of the subjects according to their kidney function.

Fourth, the subjects for longitudinal analyses had more homogenous values for 25(OH)D, PTH, and BNP than the subjects for cross-sectional analyses which make it difficult to show associations; i.e. PTH range (min, max) decreased from (1.7, 24.3) to (2.5, 14.7) (pmol/l). The smaller sample size and range of 25(OH)D and PTH could have contributed to the lack of longitudinal associations. Another explanation could be that the findings for low 25(OH)D and high PTH observed in HF patients may not be translatable to a population with nonHF subjects. Future studies could benefit from a larger sample size and a wider range of vitamin D and PTH to show potential longitudinal associations.

In our study, we observed effect modification by kidney function (eGFR) in the cross-sectional association of 25(OH)D and BNP. Subjects with low eGFR have lower kidney-specific 1α-hydroxylase activity (catalyzes hydroxylation of 25(OH)D to calcitriol, the active form of vitamin D). Decreased synthesis of calcitriol occurs already at eGFR values <90 ml/min (27). Stratification based on the eGFR, led to a stratum in which the subjects had an eGFR <60 ml/min which suggest decreased synthesis of calcitriol. Serum calcitriol levels might also be independently associated with BNP levels. Unfortunately, we did not measure calcitriol levels and are not able to draw conclusions whether different calcitriol levels would be related to BNP.

Higher BNP levels could also be affected by impaired kidney function given the important inter-relationship between cardiac and renal dysfunction. There is uncertainty about the effect of declining eGFR and accumulation of BNP concentrations. Nevertheless, recent evidence suggests that BNP does not accumulate because of moderate renal impairment but reflects cardiac production rather than impaired clearance (28, 29).

Effect modification by kidney function (eGFR) was also observed for the association between PTH and BNP. Impaired kidney function results in decreased calcitriol production and increased PTH levels. If kidneys are not able to reabsorb calcium, blood calcium levels will fall which stimulates continual secretion of PTH to maintain blood calcium levels (27). Probably, subjects having high PTH are more susceptible for the negative influence of vitamin D on cardiac health.

### Potential mechanisms

There are potential mechanisms through which low vitamin D and high PTH could harm the cardiac system. The underlying pathways are still unclear as the interdependency of vitamin D, PTH, and calcium makes it hard to study individual factors. Low 25(OH)D is associated with cardiac cell growth and hypertrophy and can thereby stimulate BNP secretion (4). Vitamin D could also be involved in the pathogenesis of arterial hypertension via stimulation of the renin–angiotensin system (30). Vitamin D deficiency may predispose to hypertension via elevation of PTH and disturbed calcium homeostasis, although the relationship between vitamin D and blood pressure is not consistent (30). In our study, adjusting for SBP did not change the associations of PTH and 25(OH)D with BNP (data not shown). Therefore, our study does not support the hypothesis that the association between 25(OH)D and heart function occurs via higher blood pressure. Vitamin D may also be cardioprotective by its anti-inflammatory effects as shown in a trial in HF patients (31). Myocardial calcium homeostasis, which is crucial for the contractility and electrophysiology of the heart, is also partially regulated by calcitriol, mediated by its influence on ion channels and enzymatic reactions (32). Therefore, sufficient vitamin D status could prevent myocardial hyper-contractility and maintain diastolic function (32).

PTH may influence intracellular signaling by an increase of intracellular calcium in the cardiomyocytes. PTH receptors have been demonstrated in the heart, and *in vitro* PTH induces hypertrophy of cardiomyocytes (33, 34). In addition, PTH activates protein kinase C which could lead to hypertrophic growth and expression of fetal type proteins in cardiomyocytes (35). This might contribute to a decline in ejection fraction and increase in BNP seen in 1 year after parathyroidectomy in patient with mild primary hyperparathyroidism (34). This hypertrophic effect of PTH may lead to increased susceptibility for cardiac diseases and increased risk for HF (35).

Strength of this study is the use of BNP as an easily obtainable and sensitive marker of early myocardial deterioration in subjects not suffering from HF. An early diagnosis of HF could be beneficial to prevent or postpone HF and cardiac diseases, if modifiable determinants are known. Our study allowed us to investigate subjects with moderately increased BNP which already indicates a higher future risk of developing cardiac diseases (8). The present findings extend the results of studies that reported associations between vitamin D and BNP within the clinical range in select patient groups, such as patients on dialyses (23, 36) or HF patients (13).

In conclusion, lower 25(OH)D was cross sectionally associated with higher BNP in subjects with impaired kidney function only (eGFR ≤60 ml/min). The association was not independent of PTH because adjusting for PTH attenuated the association. Higher PTH was cross sectionally associated with higher BNP levels in subjects with impaired kidney function, independent of 25(OH)D. These findings may point to a potential role for PTH in myocardial function, although the results should be interpreted with caution because of the design, sample size, and missing values. Confirmation by larger prospective studies is needed. No longitudinal associations of 25(OH)D and PTH were observed with BNP, which may have been due to small sample size and/or healthy selection, or the study population.

## Figures and Tables

**Table 1:** Baseline characteristics according to season- and sex-specific 25(OH)D and PTH quartiles in 502 subjects.

**Demographics**	**Serum 25(OH)D** (nmol/l)					**Serum PTH** (pmol/l)				
	Quartile 1^a^	Quartile 2^a^	Quartile 3^a^	Quartile 4^a^		Quartile 1^a^	Quartile 2^a^	Quartile 3^a^	Quartile 4^a^	
Mean 25(OH)D (range)	30.2 (15.7, 50.6)	44.3 (33.1, 67.4)	57.6 (42.4, 75.7)	78.1 (58.9, 111.2)		59.1 (16.8, 107.4)	52.6 (21.1, 106.3)	50.1 (16.7, 108.0)	47.2 (15.7, 111.2)	
Mean PTH (range)	6.8 (3.1, 24.3)	6.4 (3.0, 17.9)	6.0 (2.5, 14.7)	5.2 (1.7, 11.0)	***P* trend**	3.8 (1.7, 4.8)	5.2 (3.9, 6.2)	6.3 (5.2, 7.6)	9.2 (6.0, 24.3)	***P* trend**
*n*	129	125	127	121		122	126	130	124	
Age (years)	73.1±6.5^b^	70.4±6.8	69.1±6.2	67.0±5.3	<0.001	68.8±6.3	69.5±6.5	70.9±7.2	70.4±6.2	0.021
Females (%)	51.2	52.8	48.8	48.8	0.902	50.0	50.8	50.0	50.8	0.997
Education level										
Low	43.5	50.8	45.6	51.7		52.5	47.9	50.8	40.0	
Intermediate	37.9	39.4	44.0	35.0	0.316	34.4	42.2	35.1	45.0	0.398
High	18.6	9.8	10.4	13.3		13.1	9.9	14.1	15.0	
Prior CVD (%)	54.9	47.6	40.7	43.7	0.133	45.4	41.6	52.0	47.9	0.412
Type 2 diabetes (%)	28.9	18.9	22.2	17.4	0.051	21.4	19.8	16.2	30.6	0.039
Arterial hypertension (%)	77.3	77.6	63.0	58.3	0.001	56.6	65.1	72.9	82.1	<0.001
Lifestyle										
Cigarette smokers (%)	16.7	17.6	9.4	20.0	0.122	20.5	15.2	15.5	12.3	0.365
Physical activity (h/day)	2.7±2.1	3.3±2.7	3.2±2.5	3.6±2.5	0.006	3.1±2.2	3.6±2.4	3.1±2.8	3.0±2.4	0.539
Outdoor activities (h/day)	0.8±0.8	1.0±1.1	1.1±1.4	1.4±1.3	<0.001	1.1±1.1	1.2±1.1	1.0±1.3	1.0±1.1	0.462
Adiposity										
BMI (kg/m^2^)	27.9±4.8	27.6±3.9	27.1±3.4	26.3±3.4	0.001	26.0±3.3	27.1±3.6	27.1±3.7	28.7±4.8	<0.001
Waist circumference (cm)	97.7±12.6	95.5±11.9	94.5±10.9	91.9±11.3	<0.001	91.8±11.4	93.8±11.4	95.1±11.0	99.0±12.6	<0.001
Metabolic variables										
SBP (mmHg)	149.1±22.0	142.9±19.6	138.7±20.6	138.6±18.9	<0.001	138.9±21.0	141.4±18.8	143.5±22.5	145.7±20.0	0.007
DBP (mmHg)	84.5±12.6	84.2±11.0	81.8±10.8	81.7±9.3	0.016	80.7±10.5	82.7±10.1	82.6±11.2	86.2±11.8	<0.001
Fasting glucose (mmol/l)	6.3±1.3	6.0±1.0	6.2±1.6	6.0±1.0	0.217	6.0±1.1	6.0±0.9	6.0±0.9	6.6±1.8	0.001
Post-load glucose (mmol/l)	8.0±3.0	7.3±2.4	7.2±2.8	6.7±2.4	<0.001	6.9±2.4	7.2±2.6	7.2±2.5	7.9±3.2	0.007
HbA1c (%)	6.0±0.6	5.9±0.6	5.9±0.5	6.1±0.8	0.119	5.9±0.6	5.9±0.6	5.8±0.5	6.1±0.8	0.100
Triglycerides (mmol/l)	1.6±0.9	1.5±0.7	1.5±0.9	1.4±0.5	0.017	1.5±0.8	1.5±0.8	1.4±0.7	1.6±0.9	0.518
LDL cholesterol (mmol/l)	3.5±0.9	3.7±1.0	3.7±1.0	3.8±0.9	0.050	3.6±0.9	3.8±0.9	3.5±0.9	3.6±1.0	0.486
HDL cholesterol (mmol/l)	1.4±0.4	1.4±0.4	1.4±0.4	1.5±0.4	0.004	1.5±0.4	1.4±0.4	1.5±0.4	1.3±0.4	0.049
eGFR (ml/min)	62.4±11.0	61.8±10.4	61.4±11.2	62.2±10.4	0.820	63.1±9.9	62.3±10.3	62.7±10.0	59.6±12.3	0.020
eGFR <60 ml/min (%)	45.7	44.8	47.2	43.0	0.923	44.3	44.4	42.3	50.0	0.645
Serum creatinine (μmol/l)	93.5±17.0	94.9±14.8	97.7±18.9	97.8±20.1	0.028	94.4±14.0	95.1±14.0	94.1±15.4	100±25.4	0.019
BNP (pmol/l)^c^	14.8±22.7	11.6±14.5	9.1±7.1	10.2±9.6	0.005	8.7±8.0	9.2±6.9	13.1±18.6	14.8±20.2	<0.001

25(OH)D, 25-hydroxyvitmain D; CVD, cardiovascular diseases; BMI, body mass index; DBP, diastolic blood pressure; eGFR, estimated glomerular filtration rate; PTH, parathyroid hormone; SBP, systolic blood pressure.

a Quartiles are season- and sex-specific and therefore values may overlap.

b Data are presented as percentages and mean±s.d.

c Baseline BNP levels are adjusted by adding 2.66 pmol/l (for comparability to levels at follow-up) (17).

**Table 2:** Eight years changes of BNP and associations of baseline 25-hydroxyvitamin D quartiles with BNP after 8 years of follow-up in 278 subjects.

	**Serum 25-hydroxyvitamin D** (nmol/l)				
Mean and range (min–max)	1st Quartile 33.3 (16.7, 62.4)	2nd Quartile 46.8 (33.4, 69.1)	3rd Quartile 59.6 (45.0, 79.2)	4th Quartile 77.9 (63.4, 103.3)	*P* trend
*n*	65	73	73	67	
Absolute BNP changes (pmol/l)^a^	20.9±33.8	15.0±20.5	13.0±22.7	11.6±14.2	0.023
Regression coefficients					
Model 1	2.4 (−6.6, 11.6)^b^	−1.2 (−9.9, 7.6)	−1.7 (−10.3, 6.8)	Reference	0.593
Model 2	2.6 (−5.3, 10.4)	−2.9 (−10.4, 4.6)	−1.0 (−8.4, 6.3)	Reference	0.654
Model 3	2.4 (−5.6, 10.5)	−3.0 (−10.6, 4.6)	−1.1 (−8.5, 6.3)	Reference	0.691

BNP, B-type natriuretic peptide. Model 1, adjusted for age; model 2, model 1+adjusted for baseline BNP, smoking, outdoor activities, body mass index, triglycerides, and high density lipoprotein cholesterol; model 3, model 2+eGFR.

a Baseline BNP adjusted to follow-up BNP by adding 2.66 pmol/l to baseline BNP (17); presented as mean changes±s.d. in pmol/l.

b A positive regression coefficient implies higher BNP.

**Table 3:** Eight years changes of BNP and associations of baseline parathyroid hormone quartiles with BNP after 8 years of follow-up in 278 subjects.

	**Serum parathyroid hormone** (pmol/l)				
Mean and range (min–max)	1st Quartile 3.9 (2.5, 4.8)	2nd Quartile 5.1 (4.1, 6.0)	3rd Quartile 6.2 (4.7, 7.9)	4th Quartile 8.8 (5.8, 14.7)	*P* trend
*n*	66	72	73	67	
Absolute BNP changes (pmol/l)^a^	12.8±15.0	16.4±25.0	15.8±26.8	15.0±26.3	0.334
Regression coefficients					
Model 1	Reference	4.5 (−4.0, 13.0)^b^	2.7 (−5.8, 11.2)	4.2 (−4.4, 12.9)	0.445
Model 2	Reference	1.7 (−5.6, 9.0)	2.0 (−5.1, 9.4)	−2.5 (−10.1, 5.2)	0.556
Model 3	Reference	2.0 (−5.4, 9.3)	2.2 (−5.2, 9.6)	−2.3 (−10.2, 5.6)	0.603

BNP, B-type natriuretic peptide. Model 1, adjusted for age; model 2, model 1+baseline BNP, smoking, outdoor activities, body mass index, triglycerides, and high density lipoprotein cholesterol; model 3, model 2+eGFR, 25(OH)D.

a Baseline BNP adjusted to follow-up BNP by adding 2.66 pmol/l to baseline BNP (17); presented as mean changes±s.d. in pmol/l.

b A positive regression coefficient implies higher BNP.
